# Microstructure Effect of Heat Input on Ballistic Performance of Welded High Strength Armor Steel

**DOI:** 10.3390/ma14195789

**Published:** 2021-10-03

**Authors:** Elson Renato Santos Souza, Ricardo Pondé Weber, Sergio Neves Monteiro, Suzane de Sant’Ana Oliveira

**Affiliations:** 1 Brazilian Army Assessment Center—CAEx, Rio de Janeiro 23020-240, Brazil; elsonqmb@yahoo.com.br; 2 Department of Materials Science, Military Institute of Engineering—IME, Rio de Janeiro 22290-270, Brazil; rpweber@ime.eb.br; 3 Department of Inorganic Chemistry, Federal University of Rio de Janeiro—UFRJ, Rio de Janeiro 21941-909, Brazil; susan.oliver@hotmail.com

**Keywords:** ballistic armor, low alloy high strength steel, shielded metal arc welding (SMAW), fusion zone (FZ) and heat affected zone (HAZ), microstructure, V_50_ ballistic resistance

## Abstract

The effect of two different heat inputs, 1.2 and 0.8 kJ/ mg, on the microstructure associated with a welded high hardness armor (HHA) steel was investigated by ballistic tests. A novel way of comparing the ballistic performance between fusion zone (FZ), heat-affected zone (HAZ), and base metal (BM) of the HHA joint plate was applied by using results of the limit velocity V_50_. These results of V_50_ were combined with those of ballistic absorbed impact energy, microhardness, and Charpy and tensile strength revealing that the higher ballistic performance was attained for the lower heat input. Indeed, the lower heat input was associated with a superior performance of the HAZ, by reaching a V_50_ projectile limit velocity of 668 m/s, as compared to V_50_ of 622 m/s for higher heat input as well as to both FZ and BM, with 556 and 567 m/s, respectively. Another relevant result, which is for the first time disclosed, refers to the comparative lower microhardness of the HAZ (445 HV) vs. BM (503 HV), in spite of the HAZ superior ballistic performance. This apparent contradiction is attributed to the HAZ bainitic microstructure with a relatively greater toughness, which was found more determinant for the ballistic resistance than the harder microstructure of the BM tempered martensite.

## 1. Introduction

Steels used in ballistic armor need both a high hardness to stop an armor-piercing bullet by shattering its tip as well as enough toughness to prevent armor fragmentation after the projectile impact [1,2,3,4]. The way to simultaneously achieve these properties, which in principle are antagonists, is by combining a heat treatment, usually quench and temper (Q&T), and adding alloying elements that increase the hardenability of the material [5,6,7,8]. In the case of high hardness steel for armor (HHA), the main alloying additives are chromium (Cr), nickel (Ni), and molybdenum (Mo). In the fabrication of combat vehicles, HHA are usually welded by shield metal arc welding (SMAW) [9,10], flux core arc welding (FCAW) [5], gas metal arc welding (GMAW) [11,12], or laser-arc hybrid welding (LAHW) [13]. The welded joint, comprising the fusion zone (FZ) and heat affected zone (HAZ), has been considered over the years a relatively softer region, which should have lower ballistic resistance, in comparison to the base metal (BM). Indeed, in an earlier report, Wilson [14] stated that hardness is a major requirement for ballistic performance. Ade [15] as well as Reddy and Mohandas [16] corroborated hardness as a main factor in the ballistic qualification of armor steel weldment. In a general work on metallic armors, Crouch [17] emphasized the relevance of hardness on the ballistic performance of materials from aluminum to high strength steels. The special case of welded HHA is of common interest regarding ballistic protection. According to Ramana et al. [18], the harder the welded steel the better is its ballistic performance. Indeed, welded low alloy HHA are typically applied where weight reduction and projectile penetration resistance become key considerations [5].

However, other factors might also be relevant regarding the ballistic resistance of a low alloy HHA welded joint. The microstructure plays an important role and is associated with the mechanical properties [15]. While the BM consists of harder tempered martensite resulting from the Q&T treatment, the relatively softer FZ and HAZ depend on the welding heat input. A higher heat input (HHI) was followed by fast cooling results in coarse-grained HAZ with predominantly martensite microstructure [18]. By contrast, Pramanick et al. [19] indicated that weld joint with lower heat input (LHI) show good ballistic performance. No specific microstructure was mentioned by the authors for the HAZ. 

In addition to works focusing on microstructure, those on process conditions effects, such as different welding techniques [20]; thermal cycling [17]; filler [21]; welding consumables [22]; welded joint design [23]; protective weld coatings [24] and welding heat input [16,25], have also been conducted. As above mentioned, heat input is a welding process parameter that might significantly affect the ballistic performance of Q&T HHA. Indeed, Reddy and Mohandas [16] investigated the effect of welding heat input on the hardness and ballistic performance of a Q&T high strength low alloy (1.00 Cr, 0.75 Mn, 0.55 Si, 0.35 Mo, and 0.10 Zr) steel. They found that the width of the softened HAZ increases with the heat input. As such, a greater heat input was associated with wider and softer HAZ that promoted a poor ballistic performance. They also indicated that, for the investigated heat inputs, the microstructure of the HAZ was predominantly composed of martensite. Reddy and Mohandas [16], however, did not indicate the steel origin or supplier. Moreover, other information, such as the Q&T steel tensile strength and bullet impact energy, was not disclosed. It was also missing the interpretation of the term “ballistic limit”, used in the article [16] as an indication of ballistic performance. It is also worth mentioning that in a recent work Savic and Cabrilo [25] also investigated the effect of heat input on the ballistic performance of armor steel and found the HAZ of the LHI more prone to projectile penetration than that of the HHI using the criterion of no punch holes made upon three fired projectiles; the authors reported that the HHI weldment failed to meet this criterion. As such, the LHI was found to have a higher ballistic performance than the HHI, which was not resistant to 7.62 × 51 mm projectile penetration [25]. The same ammunition is now used in the present work.

Based on the relevant conclusions of Reddy and Mohandas [14] as well as Savic and Cabrilo [25] and motivated by the need to complement some open questions, the present work investigated the effect of heat input on the ballistic behavior of a welded Q&T high strength low alloy (Cr, Ni, Mo) HHA steel. The following questions still need to be answered. Is the HAZ microstructure only martensitic for both high and low heat inputs? If so, how does one would explain a poor ballistic performance associated with high HHI? Moreover, in all investigated Q&T HHA welded joints, both in terms of ballistic qualification [15] as well as effects of heat inputs [16,25], joint design [24], and microstructure [19], the ballistic performance was qualitatively estimated by the observed aspects of projectile impact resulting on either partial penetration or perforation. In the present work, for the first time, it is reported a quantitative ballistic performance by means of the standard V_50_ projectile limit velocity. Microhardness and Charpy tests were carried out in two heat input conditions, HHI and LHI, and the microstructure was evaluated along the FZ, the HAZ, and the BM by scanning electron microscopy (SEM). Ballistic tests were performed in the FZ, HAZ, and BM by measuring the impact energy as well as the V_50_ ballistic limit. The results of the two different heat inputs were compared between each other as well as with the BM to evaluate their influence on the ballistic performance of the relatively softer FZ and HAZ regions of the weld.

## 2. Materials and Methods

### 2.1. Materials

The material used in this work, as the base metal (BM), is a high hardness armor (HHA) steel developed and manufactured according to the MIL-DTL-46100E standard [26] by the Brazilian Villares Metals together with USIMINAS steelmakers. The steel was provided as rolled 8 mm thick plates. A heat treatment of quenching and tempering (Q&T) yielded a hardness of 480 HB (~513 HV). Table 1 presents the chemical composition of the investigated Q&T HHA steel. The weld filler metal electrode was supplied by Rio War Arsenal.

### 2.2. Welding Process

Plates with dimensions of 500 mm × 250 mm × 8 mm of beveled Q&T HHA steel were shield metal arc welded (SMAW) as per the US Army code [27] using a ferritic low hydrogen stick electrode as filler metal. Two distinct welding heat inputs, herein indicated as lower heat input (LHI) and higher heat input (HHI), were considered as the main technical variable in the present work. 

**Figure 1:**
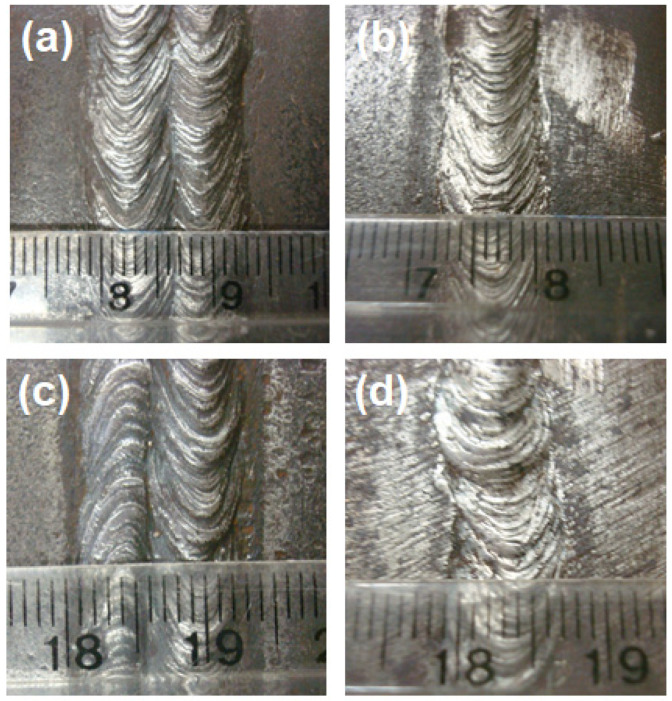
Typical Q&T HHA steel welded joints. With higher heat input: (**a**) top and (**b**) root. With lower heat input: (**c**) top and (**d**) root. (Ruler small divisions in mm).

The LHI was applied from an electrode with 2.5 mm in diameter operating with an electric current of 68.1 A, voltage of 21 V, and welding speed of 10.6 cm/min generating a heat (energy) of 0.8 kJ/mm. While the HHI was applied from an electrode with 3.25 mm in diameter operating with an electrode current of 84.7 A, voltage of 21 V, and welding speed of 8.91 cm/min, which generated a heat of 1.2 kJ/mm. Figure 1 illustrates the typical weld joints, both top and root, of the Q&T HHA steel plate for the two different conditions of LHI and HHI. In most cases welding was performed in the same direction for top and root passes, Figure 1a,b. In a few cases, reversed direction was applied in the weldment, Figure 1c,d, to verify its influence on the ballistic results.

### 2.3. Metallographic and Microstructure Analysis

Metallographic analyses were performed on samples, cut by water jet, from both the FZ and HAZ in the welded Q&T HHA steel plates. Macrostructural aspects were registered in a Zeiss stereoscope, model Stemi 2000-C and the microstructure analysis was performed by scanning electron microscopy (SEM) in a model Quanta FEG 250 FEI microscope (Schaumburg, IL, USA). 

### 2.4. Microhardness Measurements

The Vickers microhardness profile along the weld joint was obtained for both LHI and HHI welding conditions, in a Micro Vickers Hardness model Indentec Zwick Roell digital tester (São Paulo, Campinas, Brazil) with an applied load of 500 g, according to ASTM Standard [28]. Each microhardness value at a given point of the weld joint corresponds to the average of five close measurements.

### 2.5. Charpy Impact

**Figure 2:**
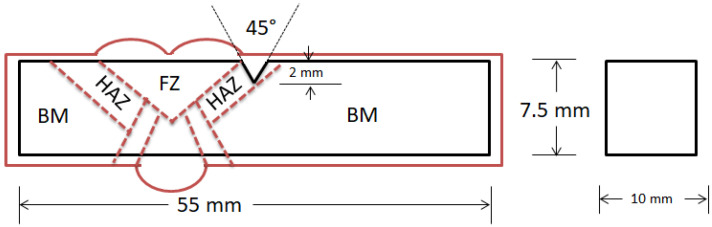
Schematic of V-notched Charpy specimen with sub-size dimensions as per ASTM E23-12c in black lines profile of the welded steel plate in red lines.

Charpy impact tests were performed in a model PW30/15R Panambra, Brazil, using an impact pendulum hammer of 30 J, to provide a preliminary evaluation of the Q&T HHA steel HHI and LHI welded joint impact toughness. V-notched specimens were machined in both longitudinal and transversal rolling direction of steel plate for the BM. As for the weldment, seven transversal specimens with V notch in the HAZ, schematically illustrated in Figure 2 and processed with either HHI or LHI, were impact tested as per ASTM E23-12c [29]. Due to the 8 mm thickness of the steel plate, non-standard sub-size dimensions of the Charpy specimen were machined as allowed by the norm.

### 2.6. Tensile Test

**Figure 3:**
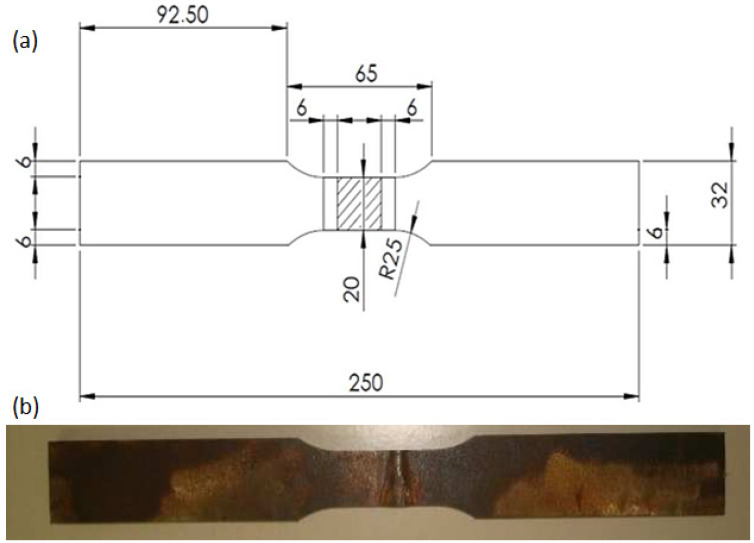
Tensile test specimens: (**a**) schematic with dimensions (mm), and (**b**) actually tested specimen with rupture beginning at the HAZ.

Tensile tests were conducted in a 100-ton Contenco and Pavitest universal machine (Minas Gerais, São José da Lapa, Brazil) as per ASTM A370-12 [30] operating with crosshead speed of 5 mm/min at room temperature. Standard flat tensile specimens were machined transversally to the steel plate rolling direction. BM specimens were cut outside the weldment. In specimens for HHI and LHI process conditions the complete weld joint was positioned at the center of the specimen gage length as shown in Figure 3. The specimen dimensions (mm) are displayed in the schematic of Figure 3a while an actual tensile tested specimen in Figure 3b revealed the beginning of rupture at the HAZ. Three specimens were tested for each condition corresponding to plain BM as well as HHI and LHI processed weldments.

### 2.7. Ballistic Testing

**Figure 4:**
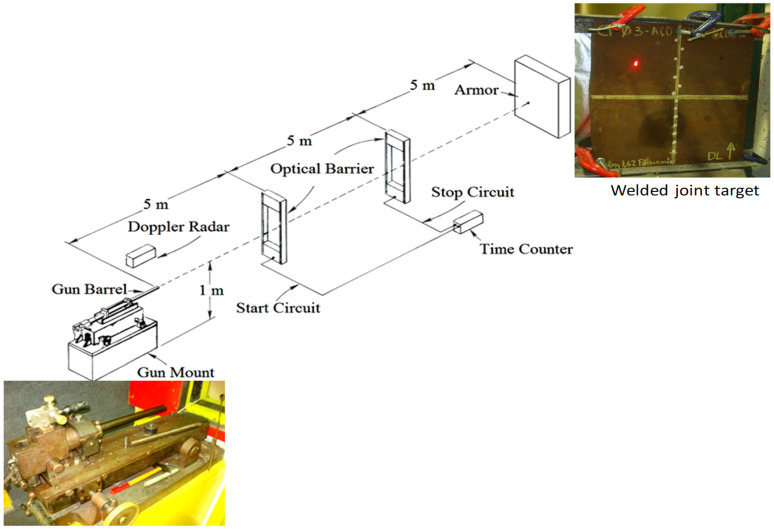
Schematic of ballistic test with actual gun barrel and welded joint target.

Ballistic tests were performed at the Brazilian Army Assessment Center (CAEx), Rio de Janeiro, according to the US Military standard [26]. A schematic illustration of the ballistic test is shown in Figure 4. Evaluation of ballistic performance was quantitatively done by the ballistic limit velocity V_50_, which provides a 50% probability of a projectile velocity to pierce a material.

**Figure 5:**
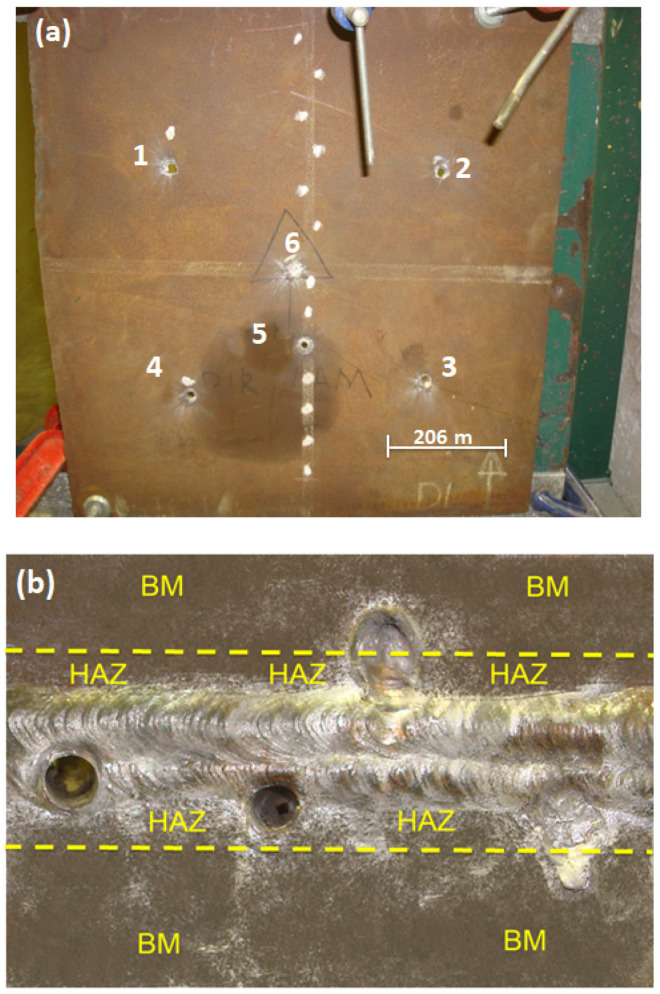
(**a**) Higher heat input Q&T HHA shield metal arc welded joint after hit by 7.62 mm bullet shootings. (**b**) Actual plate and magnification view of weldment hits. Dashed yellow line as the limit of HAZ.

According to US Military Standard [31] and US Army code [27], the ballistic limit velocity V_50_ is a method for evaluating the ballistic resistance of an armor, determined by the average value of the projectile velocity of, either 2, 3, 4, or 5 through (complete) perforations and corresponding 2, 3, 4, or 5 partial penetrations. The test ammunition was a 7.62 mm full metal jacketed projectile loaded with different amounts of powder to obtain different impact velocities and allow the ballistic velocity limit to be evaluated. The bullet weighing 9.6 g was shot from a gun barrel to a welded target sample, as shown in Figure 4. A laser sight model B 290 indicated the exact point of bullet impact, exemplified by the red bright dot in the inserted target in Figure 4. The target was placed 15 m away from the gun barrel and the bullet hit the sample at 90° with respect to its surface. The bullet velocity was measured by a model SL-520P Weiber Doppler radar (Alleroed, Denmark), provided with WinDopp^®^ software (Version 1.0) to process the radar raw data. The welded target sample was hit by the bullet with trajectory determined by the laser sight, at precisely the fusion zone (FZ) or the heat affected zone (HAZ), on welded joints fabricated with both LHI and HHI, as well as at the base metal (BM) as illustrated in Figure 5. 

An actual HHA steel plate after several hits by 7.62 mm bullet shootings is shown in Figure 5a. In this figure, shootings white-marked as 1 to 4 were performed only in the BM to assure proper ballistic properties. In particular, shooting numbers 5 and 6 are examples of weldment hits. Details with higher magnification of only weldment hits are shown in Figure 5b.

**Figure 6:**
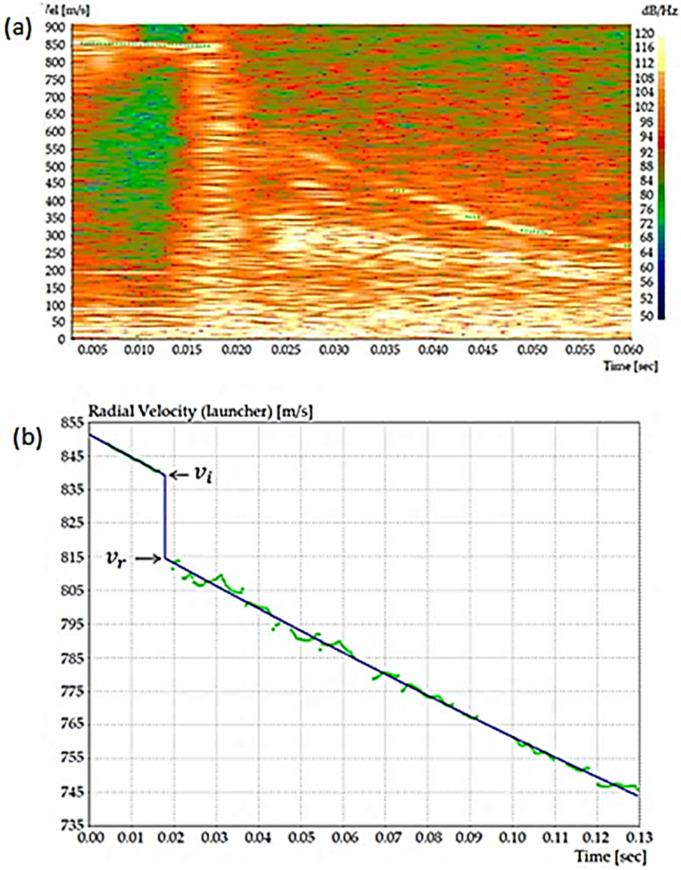
Impact and residual velocities measurement by Doppler radar: (**a**) actual computer recorded image (radar spectrum) of projectile velocity variation with time and (**b**) experimental points by FFT curve fitting. Reproduced with permission from [32].

The projectile impact velocity v_i_ against the HHA steel plate, Figure 4, and its eventual residual velocity, v_r_, leaving the plate in case of perforation are measured with precision from the Doppler radar image, as illustrated in Figure 6 from a previous work [32].

The values of both v_i_ and v_r_ were calculated using the WinDopp^®^ software, which correlates intensity with velocity by Fast Fourier Transform (FFT) to obtain the velocity versus time curve fitting, shown in Figure 6b.

Results from ballistic tests permit to measure the impact energy in addition to the ballistic limit V_50_ for each welded target sample. The impact energy E_i_ was measured by:

(1) $$E_{i}=\frac{1\;}{2}\;m{\left(v_{s}-v_{r}\right)}^{2}$$

where m = 9.6 g is the bullet mass and v_s_ the bullet striking velocity at the impact against the target sample. In case of perforation, v_r_ is the residual velocity of the bullet going out at the back of the target. The complete perforation was identified by visual observation of the passage of light in the projectile hole which is associated with the indication of a residual velocity by the Doppler radar.

## 3. Results and Discussion

### 3.1. Characterization of Base Metal Steel

The investigated Q&T HHA steel used in the present work is a Brazilian army standard, close to the AISI 4340, with yield stress of 1390 MPa, ultimate strength of 1550 MPa and total strain of 11%. Microhardness measurements on this base metal (BM) steel performed about 20 mm away from the welded joint, disclosed an average value of ~500 HV, with precise value depending on heat input, as further shown. 

### 3.2. Metallographic Analysis

**Figure 7:**
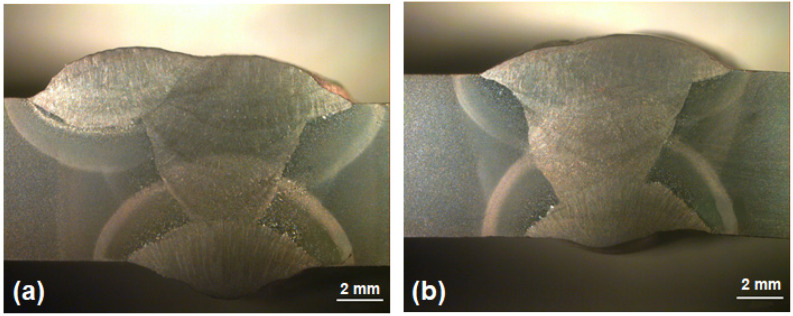
Macroscopic transversal section of welded steel: (**a**) with higher heat input; (**b**) with lower heat input.

Figure 7 presents the macrostructure of the weld joints transversal section fabricated with either high heat input (HHI) or low heat input (LHI) conditions. In this figure it should be noted that the main macroscopic difference between the two conditions is related to the width of the heat-affected zone (HAZ). The HHI welded joint, Figure 7a, is comparatively wider (~5 mm) than that (~3 mm) of the LHI, Figure 7b. These results show the influence of the heat input on the widths of the distinct HAZ regions, in which a greater heat input leads to a wider HAZ zone, corroborating the results of Reddy and Mohandas [16].

**Figure 8:**
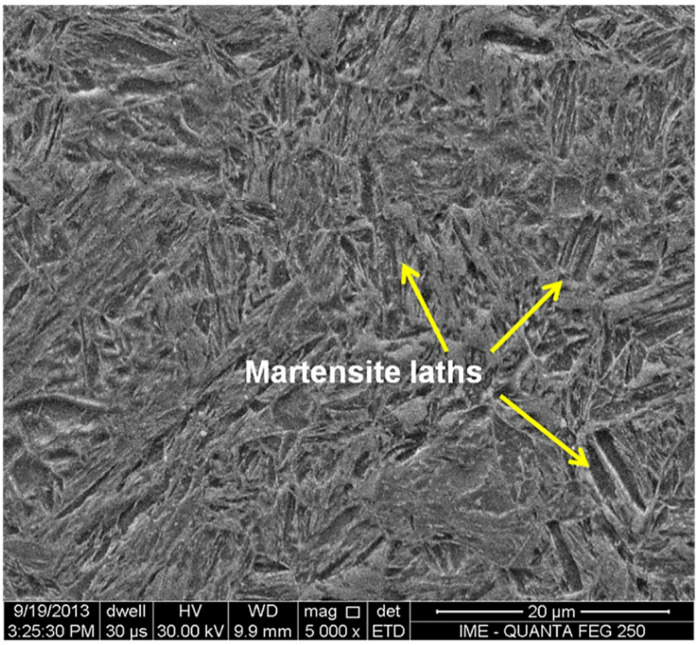
SEM micrograph of the base metal.

Figure 8 shows by SEM the typical microstructure of the BM corresponding to the as-received Q&T HHA steel. It is noteworthy in this figure that, as expected [22], the BM is mostly composed of tempered acicular martensite with evidence of martensite laths, indicated by arrows. Similar microstructure was also reported [5] for the BM of a Q&T high strength low alloy steel welded joint, which was analyzed by optical microscopy (OM) as well as SEM and transmission electron microscopy (TEM). The identification of martensite laths within the acicular martensite was made based on visually similar pictures shown by Magudeeswaran et al. [5].

**Figure 9:**
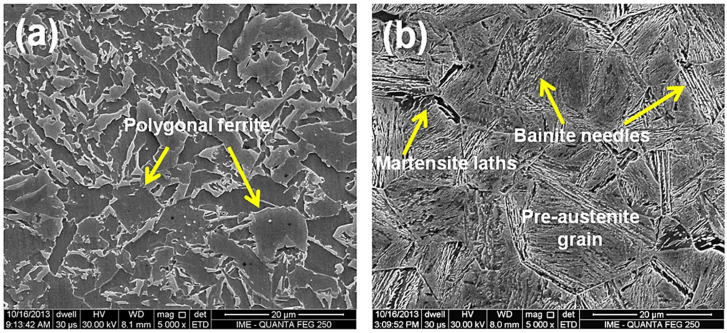
SEM micrographs of the higher heat input welded joint, in the regions (**a**) FZ and (**b**) HAZ.

**Figure 10:**
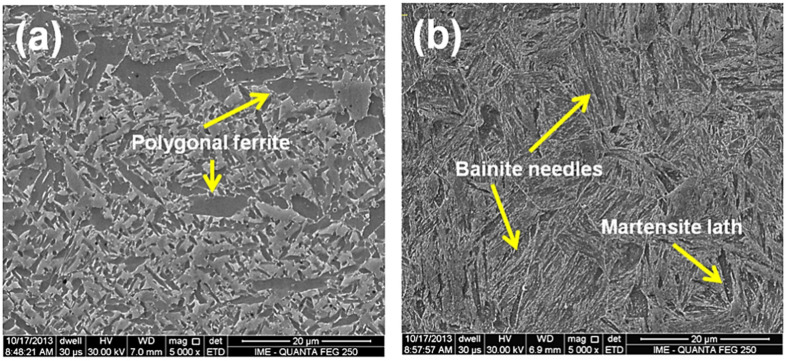
SEM micrographs of the lower heat input welded joint, in the regions (**a**) FZ and (**b**) HAZ.

Figure 9 and Figure 10 display by SEM the typical microstructures of (a) the fusion zone (FZ) and (b) the heat-affected zone (HAZ) of the Q&T HHA steel welded with HHI and LHI conditions, respectively. The FZ microstructures, on both conditions, consist predominantly of polygonal ferrite as seen in Figure 9a and Figure 10a. This is similar to the OM, SEM, and TEM results reported by Magudeeswaran et al. [5]. However, the LHI condition promotes comparatively smaller grain size (~15 µm) than the HHI (~30 µm) condition. These FZ microstructures are certainly softer than that of the BM owing to the greater grain size promoted by the HHI condition, as shown in Figure 9a.

The HAZ presents evidence of martensite laths and bainite needles that were identified in Figure 9b and Figure 10b. In particular, Figure 9b reveals the boundary of the coarsened grains of the austenite formed above AC3 temperature before the quenching heat treatment. The grain size, on both heat input conditions, Figure 9b and Figure 10b, varied with the distance from the FZ/HAZ interface, revealing that the closer to the interface, the larger the grains. The HAZ microstructure in Figure 9b for the HHI condition is composed of acicular tempered martensite and some bainite needles. The HAZ microstructure in Figure 10b for the LHI condition is composed basically of some tempered martensite laths, like those presented in the HHI condition, Figure 9b, and some acicular ferrite with predominance of bainite needles, pointed by arrows. 

The existence of bainite needles was also reported by SEM and TEM in the HAZ of a similar Q&T HHA steel (close to AISI 4340) studied by Pramanick et al. [19]. In the present work, the identification of bainite in Figure 9 and Figure 10 was based on the pictures reported and discussed by those authors. The authors concluded that among the different microstructural constituents, lower bainite with maximum toughness could be considered as the most desirable microstructure for an armor steel weld metal. Due to the importance of these constituents, Unfried et al. [33] showed a modeling approach disclosing the microstructure evolution, based on both Reddy and Mohandas [16] and Reddy et al. [20] research works.

Preliminary EDS results obtained from the SEM system failed to reveal significant difference in the distinct phases, Figure 9 and Figure 10, as compared with the steel composition in Table 1.

### 3.3. Vickers Microhardness Profile

**Figure 11:**
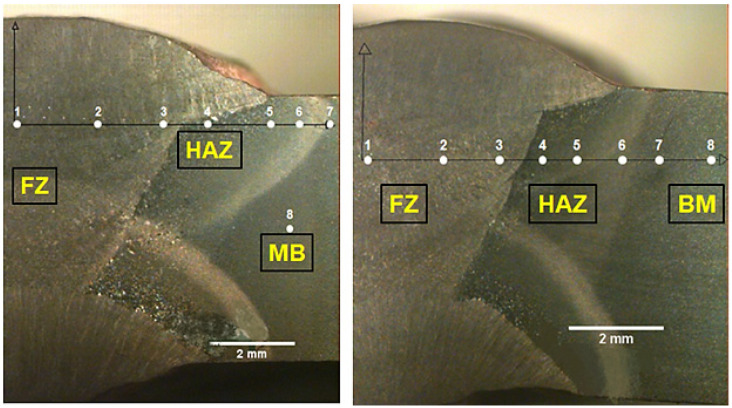
Microhardness numbered points (white) in the etched transversal section of the welded HHA steel, from the center of FZ to BM, across the HAZ. Minimum of 5 measurements per point.

**Figure 12:**
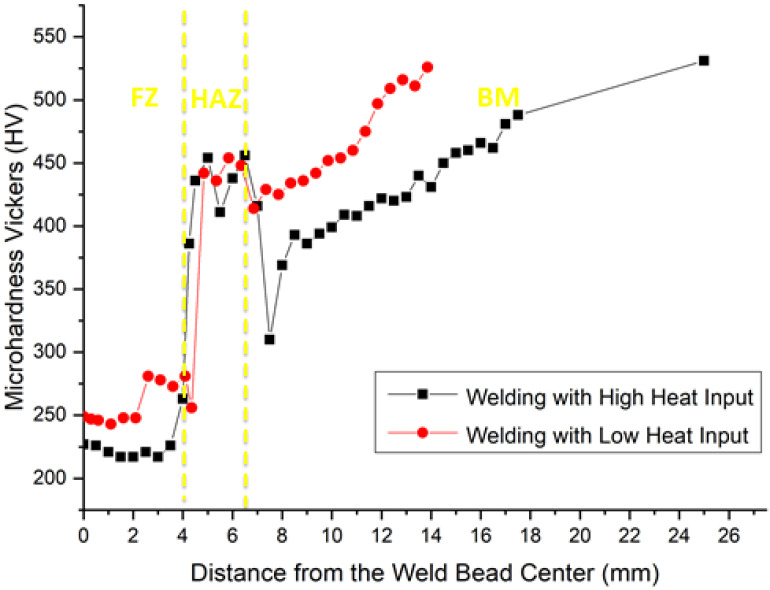
Graphical profile of microhardness measurements along the transversal section of the welded HHA steel for both heat input conditions.

Table 2 presents the microhardness values in each region, indicated by numbered white points in the etched transversal section, Figure 11, according to the graphical profile shown in Figure 12. The microhardness values in this table reveal that the LHI promoted the greatest hardness in the FZ and BM. 

According to the microstructure, Figure 11, and the microhardness profile, Figure 12, it was possible to identify, on both heat input conditions, eight different regions:

- 1st and 2nd region: with low microhardness, associated with the FZ only, where the filler metal is predominant. This region has a ductile microstructure of polygonal ferrite (219–259 HV). However, the grain size resulting from the LHI condition is smaller than that related to the HHI condition in Figure 9a and Figure 10a. Most possibly the increase in heat input contributes to growing the grains. This might be the reason why the HHI has promoted a decrease in hardness, as reported by Reddy and Mohandas [16] as well as Savic and Cabrilo [25].
- 3rd region: located close to the FZ/HAZ interface with comparatively higher hardness as a result of either coarse or thin martensite (384–275 HV). The coarse martensite was developed due to the HHI condition, Figure 9b, while thin martensite can be seen for the LHI condition in Figure 10b. For the HHI microstructure in Figure 9b, it is also possible to identify bainite needles. Generally, a microstructure composed of these bainite needles presents higher toughness [17]. However, due to the small fraction, it did not show much difference in hardness. As for the LHI condition, Figure 10b, bainite needles apparently predominate.
- 4th region in the HAZ, located close to the HAZ/FZ interface, with a sensible increase in hardness (349–430 HV), in which grows the presence of bainite needles within acicular martensite.
- 5th region inside the HAZ with characteristic predominance of bainite needles surrounding lath martensite, Figure 9b and Figure 10b, associated with a plateau of relatively high hardness (375–445 HV).
- 6th region: known as partial transformation zone located at the end of the HAZ, close to the HAZ/BM interface, showing a minor decrease in microhardness due to the presence of ferrite together with martensite, and bainite 424–429 HV);
- 7th region: identified as the BM close to the BM/HAZ interface, discloses a continuous increase in microhardness (437–446 HV) due to the lower temperatures reached in this region. It presents the same microstructure as the 6th region but with predominance of tempered martensite as well as lower percentage of bainite and ferrite.
- 8th region: corresponding to the BM only, with the highest hardness (481–503 HV) associated with tempered martensite with both acicular and lath types, Figure 8.

### 3.4. Charpy Impact Energy

Table 3 presents preliminary Charpy absorbed energy of standard V-notched specimens of the Q&T HHA BM plate in both longitudinal and transversal to the rolling direction. In this table it is also presented Charpy results of specimens machined from the weldment, transversal to the rolling direction with notch in the HAZ, Figure 2, processed with either HHI or LHI.

The results in Table 3 indicate that practically no difference should exist in absorbed impact energy regarding the direction of the base metal. However, in the weldment, the impact toughness is higher than the base metal, which was also found in a 0.27 C, 0.64 Cr, 1.09 Ni, and 0.30 Mo armor steel [34]. Moreover, the result for the weldment with LHI is superior in comparison to HHI, which is attributed to the tougher bainite [17].

### 3.5. Tensile Strength

Tensile properties related to mean values of ultimate stress and toughness, corresponding to maximum strength and area under the stress versus strain curves, respectively, are presented in Table 4 for the BM as well as HHI and LHI welded conditions. Three standard specimens, Figure 3, were tested for each condition in Table 4.

As shown in Table 4, both the tensile strength and related toughness are higher for the BM as compared to both HHI and LHI process conditions. As for the weldment, these properties for the LHI are significantly (within the standard deviation) higher than the HHI. This is coherent with the microhardness results in Table 2. However, the relatively lower value of the BM Charpy toughness in Table 3 indicates that under dynamic impact the weldment, and particularly the HAZ processed with LHI, has the highest resistance. The ballistic results confirm this performance.

At this point of our discussion, it is worth mentioning that in situations involving dynamic impact, in addition to Charpy and tensile tests, the fracture toughness might also be evaluated by other techniques such as the J-integral measurements, essential work of fracture (EWF) tests and Kahn-type tests [35] as well as finite element model and dynamic drop tests [36]. To our knowledge these techniques have not yet been applied to assess the ballistic performance of welded HHA but mainly to investigate crashworthiness in automotive high strength steels. In particular, Frómeta et al. [35] evaluated the fracture toughness of four advanced high strength steels (AHSS). They concluded that EWF technique is the most suitable parameters to describe the global fracture behavior of AHSS sheets. This technique is now included in our ongoing ballistic evaluation of welded HHA.

### 3.6. Ballistic Results

Table 5 presents the results of the V_50_ ballistic tests in the FZ and HAZ of the welds with low (LHI) and high (HHI) heat input, in addition to results in the BM. It can be noticed in this table that the ballistic limits V_50_ of the FZ for both HHI, 532 m/s, and LHI, 556 m/s, are lower than the corresponding limits of the HAZ, 622 and 668 m/s, respectively. These results indicate that the HAZ has a significantly higher ballistic resistance than that of the FZ regardless of the heat input. Moreover, welded joints fabricated with LHI have their FZ and HAZ more ballistic resistant than corresponding ones fabricated with HHI. It can also be noted in Table 5 that the FZ for both conditions, HHI and LHI, display ballistic resistance comparable to that of the BM. In fact, the FZ limit velocity for LHI (556 m/s) closely matches that of the BM (567 m/s), being only 2% below. 

This improved performance of the FZ may be related to the smaller grain size of the polygonal ferrite, Figure 9a, found with the LHI. On the other hand, the HAZ for both heat inputs display ballistic resistances superior to those of BM, 10% for HHI, and 18% for LHI. Such significant increase in ballistic resistance is possibly related to the formation of bainite that are tougher than tempered martensite. This confirms the prediction that a high toughness is necessary to obtain the best ballistic performance, which was also shown in the Charpy test results in Table 3.

The higher V_50_ of HAZ for LHI condition in Table 5 was probably obtained due to a favorable combination of hardness from the finer grain size of martensite plates in association with the toughness of bainite needles. Another parameter that corroborates the ballistic limit results is the absorbed impact energy (E_i_) presented in Table 5. It is worth noticing that for HHI, the values of E_i_ = 1.36 kJ in the FZ and 1.85 kJ in the HAZ are significantly lower than those corresponding to 1.50 kJ in the FZ and 2.14 kJ in the HAZ for LHI. Moreover, the BM also displays a mean E_i_, 1.54 kJ, significantly lower than that of HAZ associated with LHI. These E_i_ results also confirm the superior ballistic performance of the HAZ in the Q&T HHA steel welded with LHI and are supported by the microstructure results in Figure 10b. Indeed, an explanation for this superior ballistic behavior of the HAZ in welded joints fabricated with LHI is based on its microstructure.

Another point worth mentioning is that few weldments were fabricated with reversed direction regarding the two top weld beads, as illustrated in Figure 1c. The intention was to verity the influence of this procedure on the ballistic results. These few reversed welding direction experiments, within the standard deviation of all microhardness, limit velocity and absorbed impact energy, failed to show any significance difference to corresponding results of one-direction welding.

Despite the lower hardness, as compared to BM in Table 2, the HAZ of a welded Q&T HHA steel has quantitatively shown, for the first time by means of standard V_50_, to be the region with highest ballistic resistance. This is attributed to a combination of both hardness, provided by martensite, and toughness, by the bainite. Therefore, hardness is not the main factor in the steel ballistic resistance. Furthermore, the so-called soft region, associated with the FZ of the Q&T HHA steel welded joint, has a ballistic resistance comparable to that of the BM, as long as the welding process is conducted with heating input equal to or below 0.8 kJ/mm [16].

### 3.7. Ballistic Damage Mode

After ballistic test, it is required by the standard [26] to visually analyze the damage caused by the projectile impact. In principle, this damage could be ductile in association with a perforation hole with diameter close to that of the projectile and intense plastic deformation without fragmentation, or else a brittle damage with a relatively larger diameter and evidence of fragmentation due to the material’s hardness.

**Figure 13:**
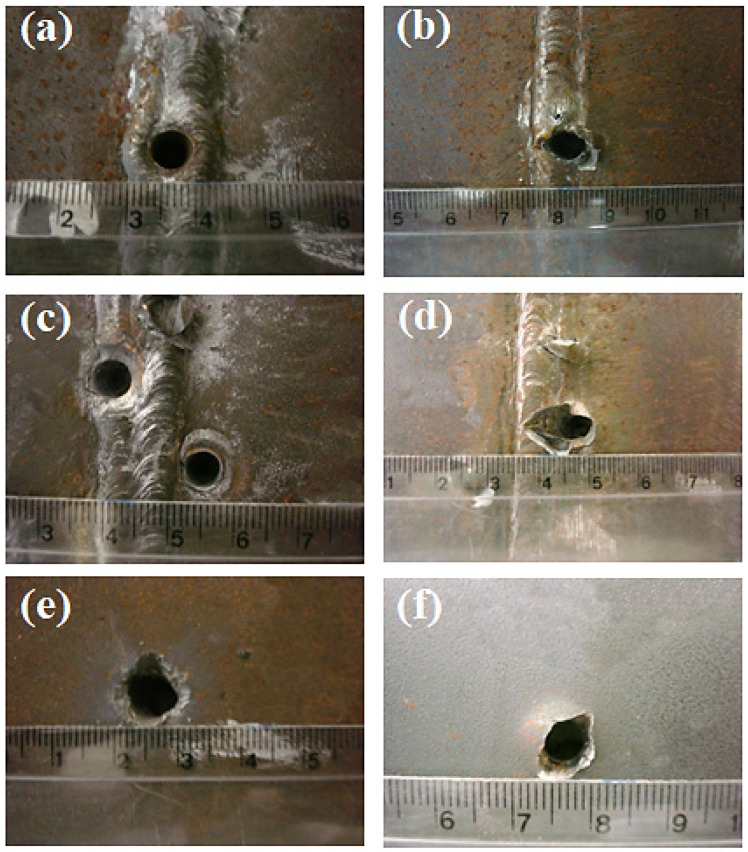
Ballistic damage at HHI weldment from 7.62 projectile impact with velocity higher than V_50_: (**a**) frontal hole in FZ; (**b**) distal hole in FZ; (**c**) frontal hole in HAZ; (**d**) distal hole in HAZ; (**e**) frontal hole in BM; and (**f**) distal hole in BM. (Ruler small divisions in mm).

Figure 13 shows the ballistic damage in the zones of the HHI processed weldment subjected to projectile velocity higher than V_50_. In this figure frontal damage at the FZ, Figure 13a, HAZ, Figure 13c and BM, Figure 13e, reveal a plugging penetration with diameter close to that of projectile and occurrence of scabbing. In addition, the corresponding distal faces, Figure 13b,d,f disclose signs of fragmentation and petaling, indicating a combination of ductile and brittle damage.

**Figure 14:**
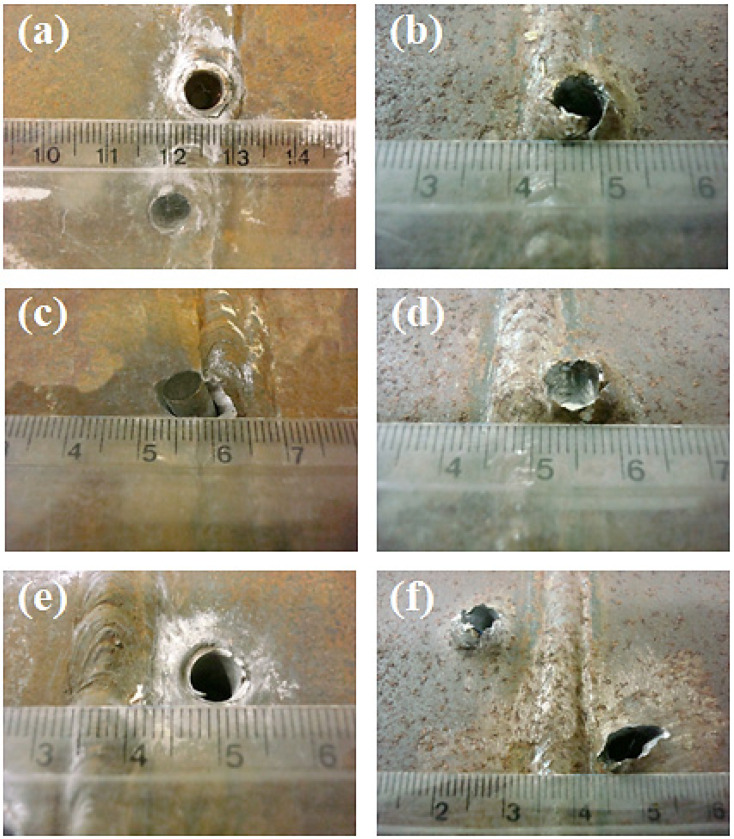
Ballistic damage at LHI weldment from 7.62 mm projectile impact with velocity higher than V_50_: (**a**) frontal hole in FZ; (**b**) distal hole in FZ; (**c**) frontal hole in HAZ; (**d**) distal hole in HAZ; (**e**) frontal hole in BM; and (**f**) distal hole in BM. (Ruler small divisions in mm).

Figure 14 shows the ballistic damage in the LHI weldment after projectile impact with velocity higher than V_50_. Similar damage modes to HHI weldment (Figure 13) are observed in this figure. In particular, a marked degree of fragmentation in the distal faces, Figure 14b,d,f might be associated with predominant brittle mode, which correlates with the higher hardness, Table 2, and impact toughness, Table 3, of LHI.

Microhardness results presented in Table 2 together with microstructure observations in Figure 9 and Figure 10, as well as ballistic limit velocity and absorbed impact energy in Table 5, confirm the superior ballistic performance of welded joints processed with LHI.

## 4. Conclusions

- The ballistic limit performance of the welded joint of a quenched and tempered (Q&T) high strength armor (HHA) steel was for the first time evaluated by the V_50_ standard method using high impact velocity 7.62 mm caliber ammunition.
- Based on two welding heat inputs; lower, 0.8 kJ/mm (LHI) and higher, 1.2 kJ/mm (HHI), selected from the literature, the effect of heat input on the hardness, considered a major ballistic requirement, was disclosed together with V_50_ for the Q&T HHA steel welded joint region.
- Quantitative results of microhardness and V_50_ limit velocity for the fusion zone (FZ), heat-affected zone (HAZ) and base metal (BM) revealed that, in spite of BM being the hardest region with 503 HV, the HAZ is ballistically the most resistant, with V_50_ = 668 m/s, associated with LHI.
- The unexpected finding that HAZ with lower hardness might be more ballistic resistant than BM is now revealed in terms of developed microstructure after the LHI welding procedure. While the BM owns its higher hardness to Q&T martensite, unaffected by the welding temperature, the HAZ displays a V_50_ ballistic tougher microstructure due to predominant formation of bainite, which causes a superior ballistic performance.
- Ballistic damage modes of HHI and LHI weldments disclosed evidence of plugging, petaling, and fragmentation associated with a combination of ductile and brittle projectile perforation. A market degree of fragmentation in the LHI distal face of FZ, HAZ, and BM indicates predominant brittle mode, which correlates with measured higher hardness and impact toughness.

## Figures and Tables

**Table 1:** Chemical composition of investigated HHA (%).

Elem	C	Mn	Si	Ni	Cr	Mo	Cu	Ti	P + S
%	0.31	0.42	0.93	0.57	0.76	0.30	0.05	0.03	0.009

**Table 2:** Microhardness at weldment regions, according to Figure 8.

Weldment	Region	High Heat Input (HV)	Low Heat Input (HV)
FZ only	1	222 ± 4.9	246 ± 2.5
	2	219 ± 2.8	259 ± 19.0
FZ near HAZ	3	384 ± 86.1	275 ± 3.5
HAZ near FZ	4	430 ± 20.3	349 ± 131.5
HAZ only	5	375 ± 33.6	445 ± 12.7
HAZ near BM	6	414 ± 6.5	429 ± 14.2
BM near HAZ	7	437 ± 13.8	446 ± 10.5
BM only	8	481 ± 26.8	24.3

**Table 3:** Charpy absorbed energy.

Notched Specimen Location	Absorbed Energy (J)
Base Metal Longitudinal	28 ± 3
Base Metal Transversal	29 ± 2
Weldment HHI	53 ± 3
Weldment LHI	69 ± 2

**Table 4:** Tensile strength and relates toughness of BM, HHI, and LHI welded condition for the Q&T HHA steel investigated.

Tensile Property	BM	HHI	LHI
Ultimate Stress (MPa)	1834 ± 58	928 ± 16	992 ± 60
Related toughness (10^3^ kJ/m^3^)	117 ± 4	82 ± 2	91 ± 5

**Table 5:** Ballistic results for the investigated HHA weldment regions. (P = partial penetration; T = through perforation).

Heat Input	Shooting	V_50_ Limit Velocity (m/s)				E_i_ absorbed Impact Energy (kJ)			
	Region								
Without heat input	Base Metal	579 (T)		565 (P)		1.61		1.53	
		557 (P)		581 (T)		1.49		1.62	
		552 (P)		568 (T)		1.46		1.55	
		V_50_ = 567				E_i_ = 1.54 ± 0.06			
High heat input	FZ	529 (P)	536 (T)		530 (P)	1.34	1.38		1.35
		531 (P)	538 (T)		546 (T)	1.35	1.39		1.43
		533 (T)	508 (P)			1.36	1.24		
		528 (P)	541 (T)			1.34	1.40		
		V_50_ = 532				E_i_ = 1.36 ± 0.05			
	HAZ	607 (P)		609 (P)		1.77		1.78	
		611 (P)		634 (T)		1.79		1.93	
		641 (T)		620 (P)		1.97		1.85	
		628 (T)		626 (T)		1.89		1.88	
		V_50_ = 622				E_i_ = 1.85 ± 0.07			
Low heat input	FZ	543 (P)	553 (P)		546 (P)	1.42	1.47		1.43
		557 (T)	547 (P)		560 (T)	1.49	1.44		1.51
		570 (T)	558 (T)			1.56	1.49		
		582 (T)	544 (P)			1.63	1.42		
		V_50_ = 556				E_i_ = 1.50 ± 0.06			
	HAZ	684 (T)		650 (P)		2.25		2.03	
		669 (T)		674 (T)		2.15		2.18	
		663 (P)		672 (T)		2.11		2.17	
		665 (P)		667 (P)		2.12		2.14	
		V_50_ = 668				E_i_ = 2.14 ± 0.06			

## Data Availability

The data presented in this study are available upon request to the corresponding author.
